# Could AI safeguard us from AI?

**DOI:** 10.1093/braincomms/fcae401

**Published:** 2024-12-13

**Authors:** Laurent Sheybani

**Affiliations:** London, UK

## Abstract

Associate Editor Laurent Sheybani discusses how artificial intelligence (AI) could be both a risk and a solution for statistical reliability in scientific research in the context of the European Union AI act, which came into force in the summer of 2024.

We all have some intuitive understanding of what artificial intelligence (AI) is—but what is it *precisely*?

According to the European law, AI is defined as ‘a machine-based system that is designed to operate with varying levels of autonomy and that may exhibit adaptiveness after deployment, and that, for explicit or implicit objectives, infers, from the input it receives, how to generate outputs such as predictions, content, recommendations, or decisions that can influence physical or virtual environments’ [Article 3 (1) of the European Union Regulation 2024/1689].^1^

If you feel that this definition is too broad, you are not alone^2^; but the broader the definition, the more likely it is to include systems that are potentially harmful without strictly being considered AI.^3^ Indeed, many have called for better security and safety of AI and, accordingly, the EU AI Act came into force last August. The Act classifies AI systems according to their risk and defines the associated requirements, which will mainly affect developers.

The objective of the Act is, on the one hand, to improve the protection and security of users and, on the other hand, to promote innovation. How will the Act impact your research? Be reassured—simply put, the Act states that the ‘Regulation does not apply to AI systems or AI models, including their output, specifically developed and put into service for the sole purpose of scientific research and development’ [Article 2 (6)].^1^ However, that does not solve the many potential scientific issues related to AI.

Beyond the legal issues related to AI, science itself will be impacted. In this Editorial, I want to discuss one particular issue related to the unprecedented ability of AI to perform numerous parallel analyses. What will be the scientist’s role when the ability of AI to analyse, test and repeat experiments exceeds that of humans? And when this happens, what will be the associated risks?

Obviously, the role that scientists will assume in this future will depend upon their field of interest, methodological approach and experimental tools. Those working in wet labs will most probably have a very different experience than those performing meta-analyses in dry labs. However, I believe that one outcome common to all scientists will be a significant increase in the ability to perform multiple analyses simultaneously. This will of course be welcomed by the community, but should also be considered with great care—are we ready to handle these multiple findings from a statistical point of view?

We should anticipate that among the many promises of AI, one is the risk to multiply analyses and experiments and thus potentially increase the risk of type I statistical errors (incorrectly rejecting the null hypothesis). Safety risks posed by AI are insufficiently investigated according to many scientists,^4^ and I believe that what could be an anecdotal risk—the impact of multiplicity on statistical reliability—could be highly detrimental to both scientific rigour and trust.

The risk of increasing type I errors will not necessarily be voluntary, which could make it even harder to pinpoint. For example, it can be shown mathematically that the more scientific teams work on the same topic, the less likely it is that the results they report are actually true.^5^ Hence, factors that are independent from the activity of a specific researcher can affect the reliability of their measures.

Do not get me wrong—I embrace the scientific opportunities promised by AI. Last year, *Brain Communications* gathered a great collection of papers that utilized AI in neurobiology and neuropathology, including a review on AI and how it can be helpful in neurology.^6^ In my field of research—epileptology—algorithms are being developed to help discriminate physiological and pathological activities,^7^ and identify interictal epileptiform discharges,^8^ which are major advances in the field. A few years ago, AI demonstrated its ability to predict protein folding,^9^ a ‘game changer’ in structural biology.^10^ More recently, the importance of AI was emphasized by the 2024 Nobel Prize in Physics and Chemistry. It would be naive and counter-productive not to take the opportunities offered by AI, and I would certainly encourage sound and interesting scientific projects that rely on or utilize AI.

So, what is the solution? Rather than viewing AI as a threat, should its rapid development in scientific research be viewed as an opportunity to rethink statistical analyses and the reliance of science on *P*-values^11^? Registered reports are studies that are accepted based on the relevance of the question and the sound methodological approach, whatever the results may be. Such a model—offered by *Brain Communications*—will certainly help to control part of the anticipated bias. Another possibility would be to look for series of ‘reliability measures’ in published literature.^5^ Indeed, specific features of articles can reflect how likely the results are to be true,^5^ just like a *P*-value. To avoid jeopardizing innovation and the discovery of unexpected results, and also because many of these metrics cannot be controlled by researchers and remain an indirect assessment of accuracy (unlike the *P*-value), my intuition would be to use such an evaluation for published literature, rather than newly submitted papers. Another challenge with such an approach is that it would be highly time-consuming and difficult to standardize, unless a method for automating the process of reading a paper, identifying specific criteria and outputting a reliability score could be identified. Come to think of it, I think I know an approach that could potentially do that...

The cover image for this issue comes from Dafinca *et al.*^12^ and shows a motor neuron differentiated from induced pluripotent stem cells of a patient with amyotrophic lateral sclerosis transduced with a GFP-expressing lentivirus.
